# TLS/FUS-ERG fusion gene in acute leukemia and myelodysplastic syndrome evolved to acute leukemia: report of six cases and a literature review

**DOI:** 10.1007/s00277-022-04979-5

**Published:** 2022-10-01

**Authors:** Heyang Zhang, Qianru Zhan, Xiaoxue Wang, Feng Gao, Jinxiang Yu, Jing Wang, Wei Fu, Pingping Wang, Xin Wei, Lijun Zhang

**Affiliations:** Department of Hematology, The First Hospital of China Medical University, Shenyang, Liaoning China

**Keywords:** t(16;21)(p11;q22), t(11;16;21)(q13;p11;q22), TLS/FUS-ERG, Acute myeloid leukemia, Myelodysplastic syndrome, Fusion gene

## Abstract

To investigate the pathogenesis and the refractory/relapse mechanisms in patients with t(16;21)(p11;q22), we retrospectively analyzed the clinical data of six cases in our hospital and sixty-two cases reported in the literature. Among the patients in our hospital, five cases were diagnosed as acute leukemia, and one was myelodysplastic syndrome evolved to acute myeloid leukemia, harboring TLS/FUS-ERG fusion gene; all the cases were detected t(16;21)(p11;q22) translocation, and five cases showed additional chromosomal abnormalities. We firstly report a novel three-way translocation t(11;16;21)(q13;p11;q22), which may affect the prognosis of leukemia with TLS-ERG fusion gene because this patient shows a more satisfactory treatment effect and deeper remission. And we found patients with TLS-ERG are more likely to have bone and arthrosis pain. Besides, CD56 and CD123 were positive in these cases, which are related to poor prognosis and the character of refractory. Moreover, some gene mutations are involved, and GATA2 and SMAD4 mutations were identified when the disease progressed from myelodysplastic syndrome to leukemia. Among sixty-two patients reported in the literature, valid positive percent of CD56 and CD123 were 81% and 14.3%, respectively. Mutation of the RUNX1 gene was detected in four cases, and one patient had multiple mutations, including BCOR, PLCG1, DIS3, BRAF, JAK2, and JAK3. The prominent feature of leukemia carrying the TLS/FUS-ERG gene is its poor prognosis. The relevant mechanism includes new mutation, jumping translocation, different transcripts, and so on. The mechanism still acquaints scarcely, which requires further study.

## Introduction

**Fig. 1:**
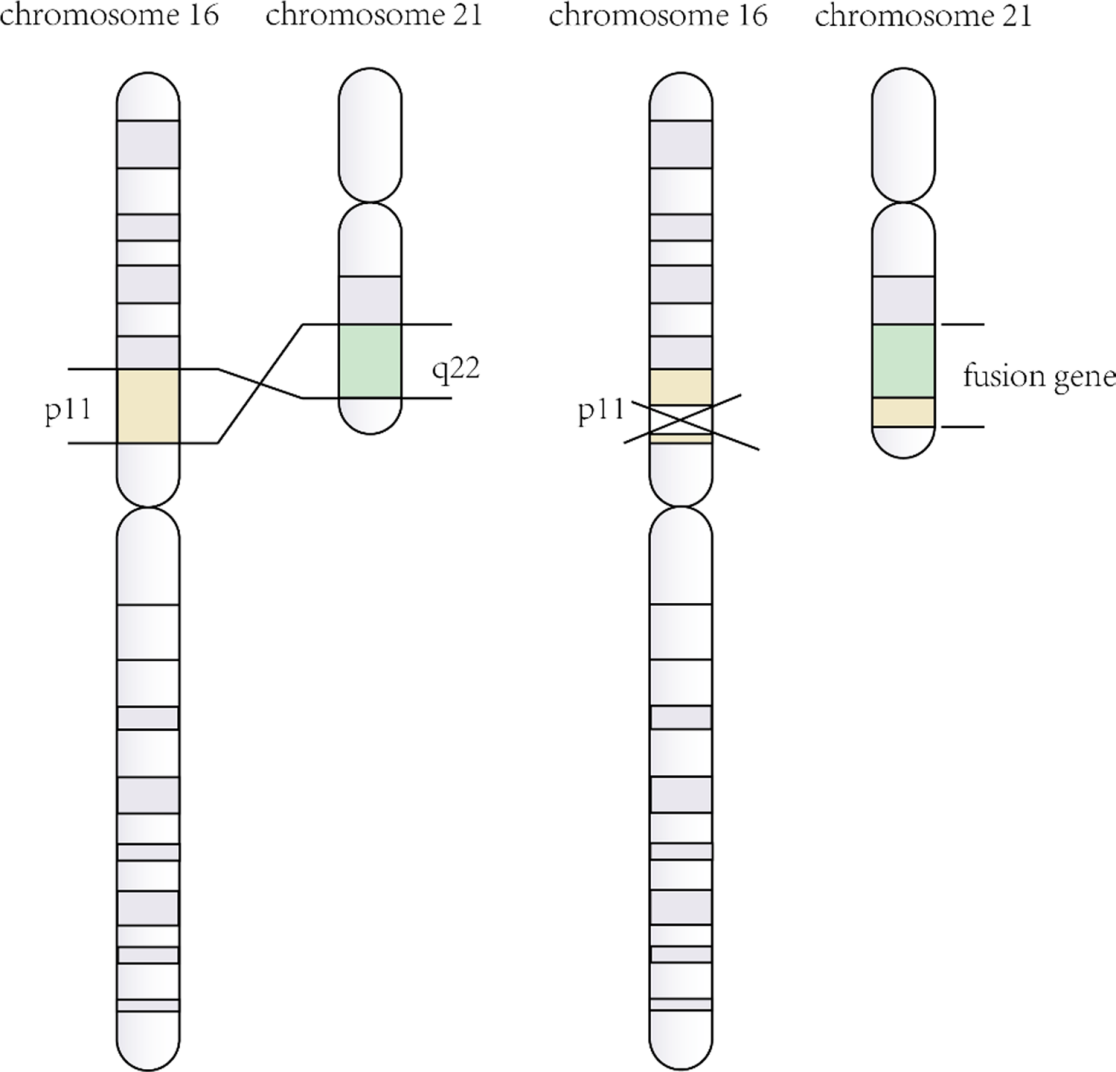
The reciprocal translocation between 16p11 and 21q22. The yellow part represents p11 on chromosome 16 and the green part represents q22 on chrosomem21. p11 is partially (or wholly) transferred to q22, contributing to the TLS-ERG fusion gene

Acute myeloid leukemia (AML) is a type of hematopoietic stem cell malignancy with highly heterogeneous, characterized by an uncontrolled clonal proliferation of abnormal myeloid stem/progenitor cells. Plenty of research has demonstrated that some fusion proteins encoded by chromosome translocations impart leukemic stem cell (LSC) properties on committed hematopoietic progenitors [1]. AML1-ETO, produced by chromosomal translocation t(8;21), acts as a driving factor in leukemogenesis [2, 3]. Additionally, it is also one of the earliest indicators used for prognostic monitoring, and provides a new strategy for therapy [4]. These findings indicate that fusion genes play an important role in leukemia and it is urgent to find a new one for further advancement of disease treatment.

t(16;21)(p11;q22) translocation is a nonrandom karyotype abnormality. This kind of chromosomal abnormality produces a fusion gene between the TLS/FUS gene at chromosome 16p11 and the ERG gene at chromosome 21q22 (Fig. 1) [5, 6]. TLS/FUS gene, which was first discovered in myxoid liposarcoma, encodes an RNA-binding protein [5]. ERG gene belongs to the ETS oncogene family, functioning as a transcriptional activator [6]. TLS/FUS-ERG is mainly reported in AML, but not in myelodysplastic syndrome (MDS) evolved to AML, acute lymphoblastic leukemia (ALL), blast crisis of chronic myelogenous leukemia (CML), and Ewing’s tumors [7–10]. In a prognostic study of 31 pediatric AML accompanied with t(16;21)(p11;q22), although morphological complete remission (CR) was 87.1%, most of the patients relapsed at an early stage and the 4-year cumulative incidence of relapse arrived at 74% [11]. In another retrospective analysis, t(16;21) or transcripts of TLS/FUS-ERG are identified as an independent poor prognostic factor among children or adolescents who were diagnosed as AML with high-risk cytogenetic abnormalities [12]. Both data show that TLS/FUS-ERG belongs to a poor prognostic subgroup.

Herein, we report six AML adult patients with TLS/FUS-ERG fusion gene and analyze the relevant clinical character. Furthermore, we reviewed the cases of TLS/FUS-ERG in the literature to get a better understanding of it (Table 1). Informed consent was obtained from all six patients.

**Table 1:** Characteristics of TLS/FUS-ERG positive cases reported in the literature

	Sex	Age	Country	FAB	WBC (× 10^9^/L)	PB blasts (%)	BM blasts (%)	Immune-phenotype	Karyotype	Gene mutation	Fusion position	Treatment	Follow-up
Panago-poulos 2013 [13]	F	2	Norway	AML	6.2	NR	50	CD13, CD33, CD15, MPO, CD7, CD56, CD34, CD117, CD133	46,XX,add(1)(p36),der(2)t(2;3)(q21;q21),del(3)(q21),der(10)t(1;10)(q32;q24),der(16)(2qter– > 2q21::16p11– > 16q24::16p11– > 16pter) [14] / 46,XX [2]	WT1	5’FUS exon 7 -ERG exon 8 3’	NOPHO-AML 2004 AIET: cytarabine, idarubicin, etoposide, thioguanine AM: cytarabine, mitoxantrone HA1M: high dose cytarabine, mitoxantrone HA2E Re-induction therapy: fludarabine, cytarabine CloEC course: clofarabine, etoposide, cyclophosphamide	PR, relapse Wait for HSCT
Koba-yashi 2017 [14]	M	2	Japan	AML	219.6	97.5	96	CD13, CD33, CD34, CD56, CD66c	49,XY, + 10,t(16;21)(p11.2;q22), + 21, + 22	NR	NR	NR	NR
	M	4	Japan	AML	7.7	NR	40	CD13, CD33, CD34, CD56, HLA-DR, CD66c	46,XY, + 10, t(2;11)(p21;p15), t(16;21)(p11.2;q22)	NR	NR	NR	NR
	M	15	Japan	AML	80.6	95	86	CD13, CD33, CD34, CD56, HLA-DR, CD66c, CD25	46,XY,t(5;6)(q31;q25),der(11)t(1;11)(p21;q13),add(21)(q22)	NR	NR	NR	NR
Kim 2009 [15]	M	36	Korea	AML	6.32	40	81	CD13, CD33, CD45, MPO; partially positive for CD34	45,XY,-16,der(21)t(16;21)(p11.2;q22) [16]	NR	5’FUS exon 7 -ERG exon 9 3’	IA: idarubicin, cytarabine (2 cycles) high dose cytarabine (2 cycles) PBSCT	CR, PBSCT > 15 months
	F	70	Korea	AML-M2	124.2	96	68.7	CD13, CD33, CD45, CD34, MPO	46,XX,dup(1)(q21q42),t(16;21)(p11.2;q22) [14] /46,XX,der(7) t(1;7)(q21;q35),t(16;21)(11.2;q22) [8]	NR	5’FUS exon 7 -ERG exon 9 3’	IA: idarubicin, cytarabine	Dead
Okoshi 2001 [17]	F	29	Japan	AML-M1	161.2	99	NR	CD13, CD33, CD34, CD56, MPO	47,XXXc, t(16;21)(p11.2;q22.3)	NR	NR	Induction chemotherapy: enocitabine, daunorubicin, 6-mercaptopurine prednisolone Re-induction chemotherapy: high dose cytosine arabinoside, idarubicin; HSCT Re-induction chemotherapy: high dose cytosine arabinoside, idarubicin;	CR, cytogenetic relapse, CR, HSCT Relapse and dead
Jekarl 2010 [16]	F	38	Korea	AML-M1	25.5	85	43	CD56 (40.2%)	45,XX,-16,der(21) t(16;21)(p11.2;q22) [16]	NA	NR	Chemotherapy HSCT	Relapse and dead 14 months
	M	37	Korea	AML-M1	44.4	95	82	CD56 (7.8%)	46,XY,t(16;21)(p11.2;q22),16qh + [15] / 46,idem,t(3;6)(q29;q21),der(13)t(1;13)(q21;q34) [4]	NA	NR	Chemotherapy PBSCT	Alive 11 months
	F	21	Korea	AML-M1	18.5	96	89	CD56 (38%)	46,XX,der(5)t(1;5)(q12;q35),t(16;21)(p11.2;q22) [18] /46,sl,t(1;7)(p32;p22) [2]/ 46.sl,del(6)(q23) [2] /48,XX, + 7, + 8,t(16;21)(p11.2;q22) [6]	RUNX1 (WT)	NR	Chemotherapy allo-HSCT	Relapse and dead 12 months
	F	59	Korea	AML-M1	6.82	87	89	CD56 (76%)	46,XX,inv(14)(q13q24),t(16;21)(p11.2;q22) [19] /46,idem,del(6)(q21q23) [2]	RUNX1 (R174Q)	NR	Chemotherapy	Relapse Alive 2 months
	M	60	Korea	AML-M5a	5.57	53	69	CD56 (87%)	46,XY,del(13)(q12q22),t(16;21)(p11.2;q22) [16]	NA	NR	Chemotherapy	Relapse and dead 11 months
	F	20	Korea	AML-M1	126	96	89	CD56 (45%)	46,XX,t(16;21)(p11.2;q22) [16]	NA	NR	Chemotherapy HSCT	Relapse and dead 18 months
	F	42	Korea	AML-M1	30.5	93	85	CD56 (85%)	46,XX,t(16;21)(p11.2;q22) [16]	NA	NR	Chemotherapy	Relapse and dead 10 months
	F	30	Korea	AML-M4	2.65	21	75	CD56 (75%)	46,XX,t(16;21)(p11;q22) [20] /46,XX [1]	NA	NR	Chemotherapy allo-HSCT	Relapse and dead 16 months
	M	41	Korea	AML-M1	29.1	91	83	CD56 (65%)	46,XY,t(16;21)(p11.2;q22) [3] /46,XY, + 1,del(1)(p12),-16, der(21)t(16;21)(p11.2;q22) [21]	NA	NR	Chemotherapy	Relapse and dead 7 months
	F	38	Korea	AML-M1	17.4	67	68	CD56 (25%)	46,XX,t(16;21)(p11.2;q22) [16]	NA	NR	Chemotherapy	Alive 2 months
	M	25	Korea	AML-M1	4.5	61	54	CD56 (20%)	46,XY,t(16;21)(p11;q22) [16]	NA	NR	Chemotherapy PBSCT	CR, relapse and dead, 19 months
	M	31	Korea	AML-M1	28.2	91	85	CD56 (45%)	46,X,idic(Y)(q12) × 2,dup(1)(q12q42),-16,der(21)t(16;21)(p11.2;q22) [7] / 47,idem, + idic(Y) [14]	RUNX1 (WT)	NR	Chemotherapy PBSCT	Dead Alive 2 months
Ismael 2014 [22]	F	2	Japan	AML-M1	39.2	NR	NR	CD13, CD33, HLA-DR	46,XX,t(16;21)(p11;q22) [8]	NA	NR	ECM regimen VP16, Ara-C, idarubicin HSCT	CR, HSCT > 121 months
	M	2	Japan	AML-M7	2.7	NR	NR	CD56, CD13, CD33, CD15, CD34, MPO, HLA-DR	46,XY,t(16;21)(p11;q22) [8] /46, idem, add(11)(q13), del(13) (q12q14) [6]	RUNX1 (at relapse)	NR	ECM regimen VP16, Ara-C, idarubicin HSCT	CR, HSCT, relapse, HSCT, relapse and dead 27 months
	F	10	Japan	AML-M1 with MLD	1.9	NR	NR	CD56, CD13, CD33, CD15, CD34, MPO, HLA-DR	45,XX,der(1;17), + 8 [2]	NA	NR	ECM regimen VP16, Ara-C, idarubicin HSCT	CR, HSCT, relapse, HSCT, dead of hepatitis 45 months
Yao 2019 [23]	M	25	China	AML-M2	NR	39	62.2	CD34, CD38, HLA-DR, CD13, CD33, CD15, CD64, CD11b, CD56, CD117, CD123, MPO, CyCD3	46,XX,t(4;8) (q28;q24.1,t(16;21) (p11.2;q22) [16] /46, XY [1]	NR	NR	DA: daunorubicin, cytarabine MA: mitoxantrone, cytarabine MA: two cycles IA: idarubicin, cytarabine; allo-HSCT; DCAG: decitabine, cytarabine Aclacinomycin, G-CSF DMA: decitabine, mitoxantrone, Ara-c CLAG: cladribine, Ara-c, G-CSF combined with donor lymphocyte infusion (DLI) CART123	CR, HSCT and relapse
Ouyang 2016 [24]	M	54	China	AML-M4	31.2	NR	NR	CD117, CD13, CD33, CD34, CD56, et al	46,XY,t(16;21)(p11;q22) [15]/45,XY,idem, − 11 [2] /47,XY,idem, + der(4)t(1;4)(q10;q10) [1] /48,XY, + X, + l, + 2, + 7, + 9, − 16, − 17, + 19, − 22 × 2 [1]	NR	NR	Induction chemotherapy Consolidation chemotherapy	CR, relapse and dead 10 months
	F	35	China	AML-M1	28.8	NR	NR	CD117, CD13, CD33, CD34, CD56, et al	46,XX,del(6)(q2l),t(16;21)(p11;q22) [10]	NR	NR	Induction chemotherapy Consolidation chemotherapy	CR, relapse and dead 27 months
	F	22	China	AML-M2	1.6	NR	NR	CD117, CD13, CD33, CD34, CD56, et al	46,XX,t(16;21)(p11;q22) [5]	NR	NR	Induction chemotherapy Consolidation chemotherapy allo-HSCT	CR Alive > 46 months
	F	38	China	AML-M1	16.7	NR	NR	CD117, CD13, CD33, CD34, CD56, et al	46,XX,t(16;21)(p11;q22) [1] /50,idem, + 4, + 10, + 15, + 22 [8] /52,idem, + 4, + 8, + 10, + 10, + 15, + 22 [1]	NR	NR	Induction chemotherapy Consolidation chemotherapy allo-HSCT	CR Alive > 11 months
	F	24	China	AML-M2	2.8	NR	NR	CD117, CD13, CD33, CD34, CD56, et al	46,XX,t(16;2 l)(p11;q22) [8]	NR	NR	Induction chemotherapy Consolidation chemotherapy allo-HSCT	Relapse and dead 28 months
	M	31	China	AML-M5	17.2	NR	NR	CD117, CD13, CD33, CD34, CD56, et al	46,XY,t(16;21) (p11;q22) [25]	NR	NR	Induction chemotherapy Consolidation chemotherapy allo-HSCT	CR alive > 24 months
	M	23	China	ANL-M2	37.2	NR	NR	CD117, CD13, CD33, CD34, CD56, et al	46,XY,der(14)t(1;14)(q10;q32),t(16;21)(p11;q22) [12] /46,XY,der(3)t(1;3)(q21;q27) [1]	NR	NR	Induction chemotherapy Consolidation chemotherapy allo-HSCT	CR, relapse and dead 21 months
	F	56	China	AML-M2	1.1	NR	NR	CD117, CD13, CD33, CD34, CD56, et al	46,XY,t(16;21) (p11;q22) [16]	NR	NR	Induction chemotherapy Consolidation chemotherapy allo-HSCT	CR, relapse and dead 12 months
	M	33	China	AML-M5	3.2	NR	NR	CD117, CD13, CD33, CD34, CD56, et al	46,XY,t(16;21) (p11;q22) [18]/46,XY − 16, + 18,21q + [5]	NR	NR	Induction chemotherapy Consolidation chemotherapy allo-HSCT	CR, relapse and dead 17 months
Kong 1997 [26]	F	11	Japan	AML-M2	14.7	30	24.8	NR	46,XX,t(16;21) (p11;q22)del(7)(q32)	NR	NR	Chemotherapy	Relapse and dead 24 months
	M	10	Japan	AML-M7	2.9	NR	NR	NR	46,XY,t(16;21) (p11;q22)	NR	NR	Chemotherapy	CR, relapse and dead 18 months
	F	2	Japan	AML-M1	39.2	62	56.5	NR	46,XX,t(16;21) (p11;q22)	NR	NR	Chemotherapy HSCT	CR and relapse, HSCT > 38 months
	F	41	Japan	AML-M1	44.2	78	90.1	NR	46,XX,t(16;21) (p11;q22)	NR	NR	Chemotherapy	Relapse and dead 16 months
	F	22	Japan	AML-M2	12.4	52	NR	NR	46,XX,t(16;21) (p11;q22)	NR	NR	Chemotherapy	CR, relapse and dead 10 months
	F	22	Japan	AML-M2	2.4	86	92	NR	46,XX,t(16;21) (p11;q22)	NR	NR	Chemotherapy	CR, relapse and dead 10 months
	F	42	Japan	AML-M2	0.8	18	65.1	NR	46,XX,t(16;21) (p11;q22)	NR	NR	Chemotherapy	CR, relapse and dead 12 months
	F	9	Japan	AML-M4	1.9	8	55	NR	46,XX,t(16;21) (p11;q22) t(9; 16),t(2; 18) del(1)(p13), + del(1)(p13), der(14)t(1; 14)(p11; p11.2)	NR	NR	Chemotherapy	CR, relapse and dead 26 months
	F	24	Japan	AML-M5a	18.7	83	86	NR	46,XX,t(16;21) (p11;q22)	NR	NR	Chemotherapy	Relapse and dead 15 months
	F	14	Japan	AML-M5b	19.9	85	89.4	NR	46,XX,t(16;21) (p11;q22)	NR	NR	Chemotherapy HSCT	CR, HSCT and dead 16 months
	M	6	Japan	AML-M7	102.0	NR	NR	NR	46,XY,t(16;21) (p11;q22),del(7q)	NR	NR	Chemotherapy	CR, dead 16 months
	M	11	Japan	AML-M7	11.8	NR	NR	NR	46,XY,t(16;21) (p11;q22)	NR	NR	Chemotherapy	CR, dead 13 months
	M	12	Japan	AML-M1	6.9	74.5	73.6	NR	46,XY,t(16;21) (p11;q22),t(1; 16)(q12; q13), t(6; 12)(q21; q13)	NR	NR	Chemotherapy	Relapse and dead 33 months
	M	46	Japan	AML-M1	120.3	91.4	91	NR	46,XY,t(16;21) (p11;q22)	NR	NR	Chemotherapy	Dead 12 months
	F	25	Japan	AML-M2	4.8	42	80	NR	46,XX,t(16;21) (p11;q22), + X/ + X, + 8	NR	NR	Chemotherapy	Relapse and dead 13 months
	M	42	Japan	AML-M2	1.9	80	NR	NR	46,XY,t(16;21) (p11;q22)	NR	NR	Chemotherapy	Dead 13 months
	F	23	Japan	AML-M5a	2.3	54	90	NR	46,XX,t(16;21) (p11;q22), + 1q	NR	NR	Chemotherapy	Dead 16 months
	M	39	Japan	AML-M5b	103.4	NR	NR	NR	46,XY,t(16;21) (p11;q22)	NR	NR	Chemotherapy	Dead 17 months
	M	61	Japan	AML-M5b	11.2	70	20.8	NR	46,XY,t(16;21) (p11;q22)	NR	NR	Chemotherapy	Dead 6 months
Harigae 1997 [27]	M	25	Japan	AML-M4	NR	NR	NR	NR	46,XY,2q^+^,9q^−^,11q^+^,t(16;21)(p11;q22)	NR	NR	Induction chemotherapy Several courses of chemotherapies Intrathecal injection PBSCT	Relapse and survive, 200 days post PBSCT
Dai 2019 [28]	M	10	Japan	AML-M5a	127,000/μL	94.5	71.8	MPO, CD11b, CD13, CD33, CD34, CD38, CD56, CD58, CD64, CD99, CD117	46,XY,del(6) (q21),t(16;21)(p11.2;q22),der(17)t(1;17)(q12;q25) [16]	NR	NR	AML‐05 protocol of the JPLSG PBSCT Azacytidine (8 cycles) low‐dose Ara‐C, aclarubicin, G-CSF, triple intrathecal therapy Cranial irradiation Fludarabine, cytarabine, L‐PAM, low‐dose total body irradiation with HLA‐matched CBT PBSCT	CR, PBSCT and relapse CR2, relapse dead of pneumonia
Jin 2019 [29]	F	55	China	AML-M5b	35.2	NR	86	CD33, CD13, CD123, CD34, CD9, MPO; medium express CD117, CD38, CD11b, CD64, CD56; weak express HLA-DR	46,XX, + 1,der(16) der(1:16)(q10;p10) t(16;21)(p11;q22), der(21)t(16;21)(p11;q22)	BCOR, PLCG1, DIS3, BRAF, JAK2, JAK3	NR	IA (idarubicin, cytarabine)	CR
Saucedo-Campos 2020 [30]	F	13	Mexico	AML-M6	20,900/mL	NR	82.5	CD10, CD19, CD22, CD79a, CD38, CD3, CD7, CD3cy, CD13, CD14, CD15, CD33, CD117, HLA-DR, CD56, CD34	NR	NR	NR	Induction chemotherapy: cytarabine, etoposide, 6-MP, doxorubicin; Maintenance therapy: 3 cycles	PR, relapse Alive 11 months
Woong 2009 [31]	M	24	Korea	AML-M5	7700/ul	84	58	CD13, CD14, CD33, CD34, HLA-DR	46,XY,t(16;21)(p11;q22),del(18)(p11.2)	NR	NR	IA (idarubicin, cytarabine) (2 cycles); HSCT	CR, relapse, dead 9 months
	M	72	Korea	AML-M0	2300/ul	29	48	CD13, CD33, CD34, CD61, HLA-DR	45,XY, -16,add(21)(q22)	NR	NR	NR	NR
Seung 2010 [32]	M	14	Korea	AML	42,680/μL	78	71	MPO, CD13, CD33, CD56	46,XY,t(16;21)(p11.2;q22) [7]/ ∼50,idem,add(1)(p13),add(1)(p21), + del(1)(q21), + del(1)(q42),del(3)(p21), + der(1;7)(q10;p10),-7, + 8, + 15,-16,der(21)t(16;21), + 22, + mar[cp13]/46,XY [1]	NR	5’FUS exon 7 -ERG exon 9 3’	NR	NR
	NR	8 mon-ths	Korea	B-ALL	46,330/μL	58	NR	CD10, CD19, CD20, CD22, HLA-DR, and TdT	45,XY,-16, der(21)t(16;21)(p11.2;q22) [10] /46,XY [10]	NR	5’FUS exon 7 -ERG exon 6 3’	Induction chemotherapy: daunorubicin, vincristine Consolidation chemotherapy	CR, relapse
Takashi 2005 [8]	M	1	Japan	B-ALL	11.5	86	NR	CD10, CD19, CD20, HLA-DR, CD13, CD33	46,XY, t(16;21)(p11;q22)	NR	5’FUS exon 7 -ERG exon 6 3’	L99-15 ALL protocol Prednisolone, vincristine, pirarubicin, L-asparaginase	NR
Coccé 2015 [33]	M	6	Argentina	ALL	6.1	13	81	CD79a, CD22, CD19, CD10, HLA-DR; partially positive for TdT, CD34, CD117	46,XY,t(16;21) (p11.2;q22) [10] /46,XY [10]	NR	5’FUS exon 7 -ERG exon 6 3’	ALL-BFM ALL-IC 2009 Induction chemotherapy: prednisone, vincristine, daunorubicin, L-asparaginase Consolidation chemotherapy: cytarabine, cyclo- phosphamide, mercaptopurine, high-dose methotrexate Re-induction therapy: dexamethasone, vincristine, doxorubicin, L-asparaginase, cyclophosphamide, cytarabine, and thiopurine Maintenance therapy: mercaptopurine and methotrexate	Free of leukemia 31 months
Toda 2017 [34]	M	65	Japan	ABL	73.77	91.2	95	CD13, CD33, CD34, CD117, CD11b^dim^, CD25, CD45RA^dim^, CD45RO, CD56, CD123; partially positive for CD203c	45,XY,?16,der(21) t(16;21)(p11;q22) [12]	NR	5’FUS exon 7 -ERG exon 10 3’	IA: idarubicin, cytarabine MEC: mitoxantrone, etoposide, cytarabine; (3 cycles) HSCT	CR, HSCT alive 14 months

*AML*, acute myeloid leukemia; *ALL*, acute lymphoblastic leukemia; *ABL*, acute basophilic leukemia; *MLD*, multilineage dysplasia; *HSCT*, hematopoietic stem cell transplantation; *PBSCT*, peripheral blood stem cell transplantation; *CBT*, cord blood transplantation; *PB*, peripheral blood; *BM*, bone marrow; *NA*, not available; *NR*, not report; *CR*, complete remission; *PR*, partial remission

## Case presentation

### Case 1

**Fig. 2:**
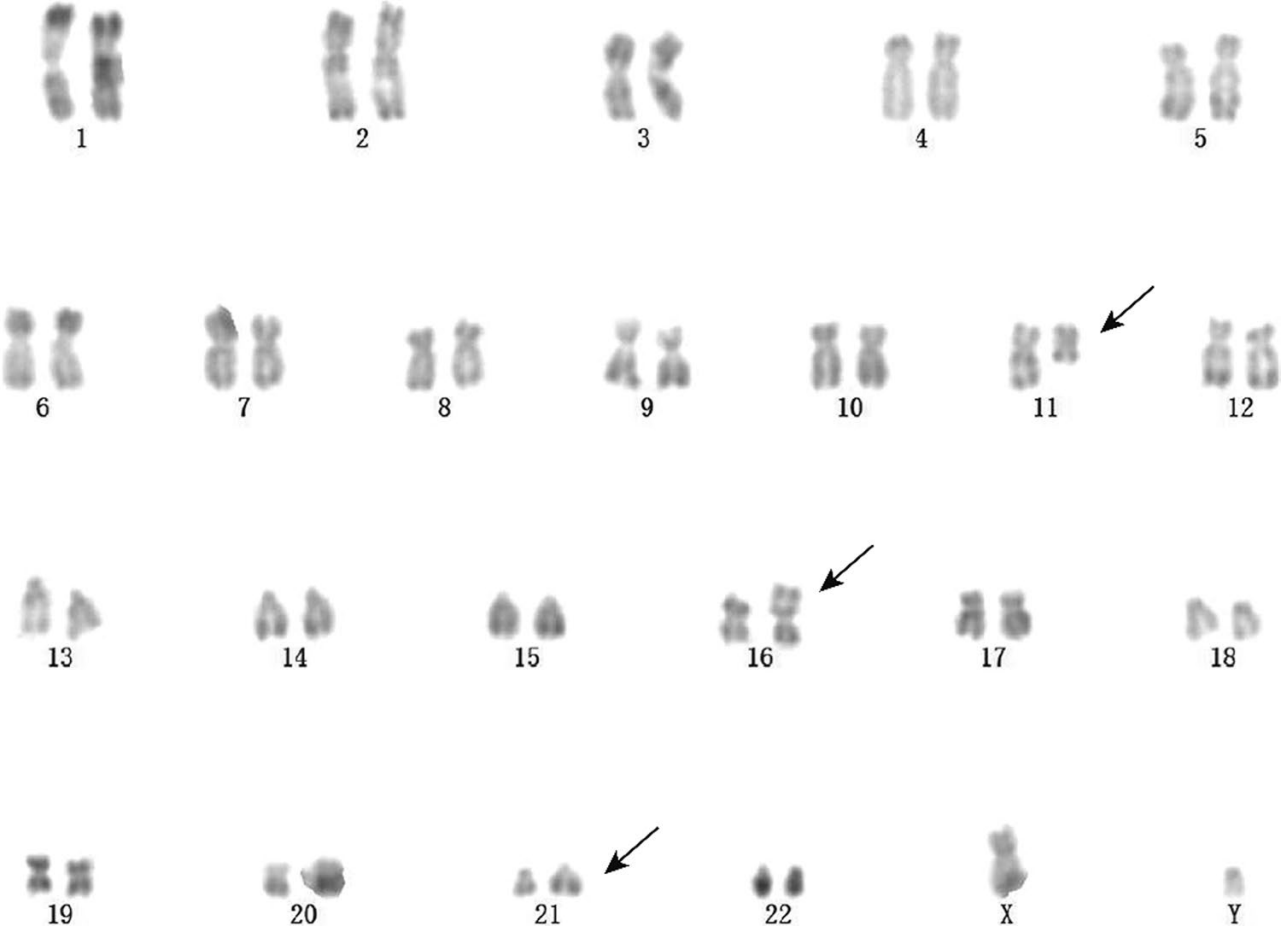
Chromosomal abnormality. The karyotype result was 46,XY,t(11;16;21)(q13;p11;q22) [16] of case 1

A 52-year-old-male was admitted to our hospital because of intermittent nosebleed and gingival bleeding in May 2021. The blood routine examination showed a white blood cell (WBC) count of 21.06 × 10^9^/L with 93.9% of blasts, a hemoglobin (Hb) level of 86 g/L, and a platelet level of 31 × 10^9^/L. The bone marrow (BM) aspirate revealed 58.0% of the primitive myeloblasts. Flow cytometry showed that the malignant immature cells accounted for 80.2%. And these cells mainly expressed CD117, CD34, and CD33; partially expressed CD56, CD13, CD38, CD123, and MPO. The karyotype result was 46,XY,t(11;16;21)(q13;p11;q22 (Fig. 2), and the TLS-ERG fusion gene was detected through RT-PCR. In addition, next-generation sequence (NGS) results showed no abnormalities. He was diagnosed with AML-M2a and an induction chemotherapy DA (daunorubicin, cytarabine) was given. For the next four sessions, the BM aspirate suggested CR at the level of morphology, immunology, and molecular biology, and the patient received four courses of medium-dose cytarabine. At present, the patient is receiving follow-up treatment at a local hospital. But the patient refused to consider the hematopoietic stem cell transplantation (HSCT).

### Case 2

**Fig. 3:**
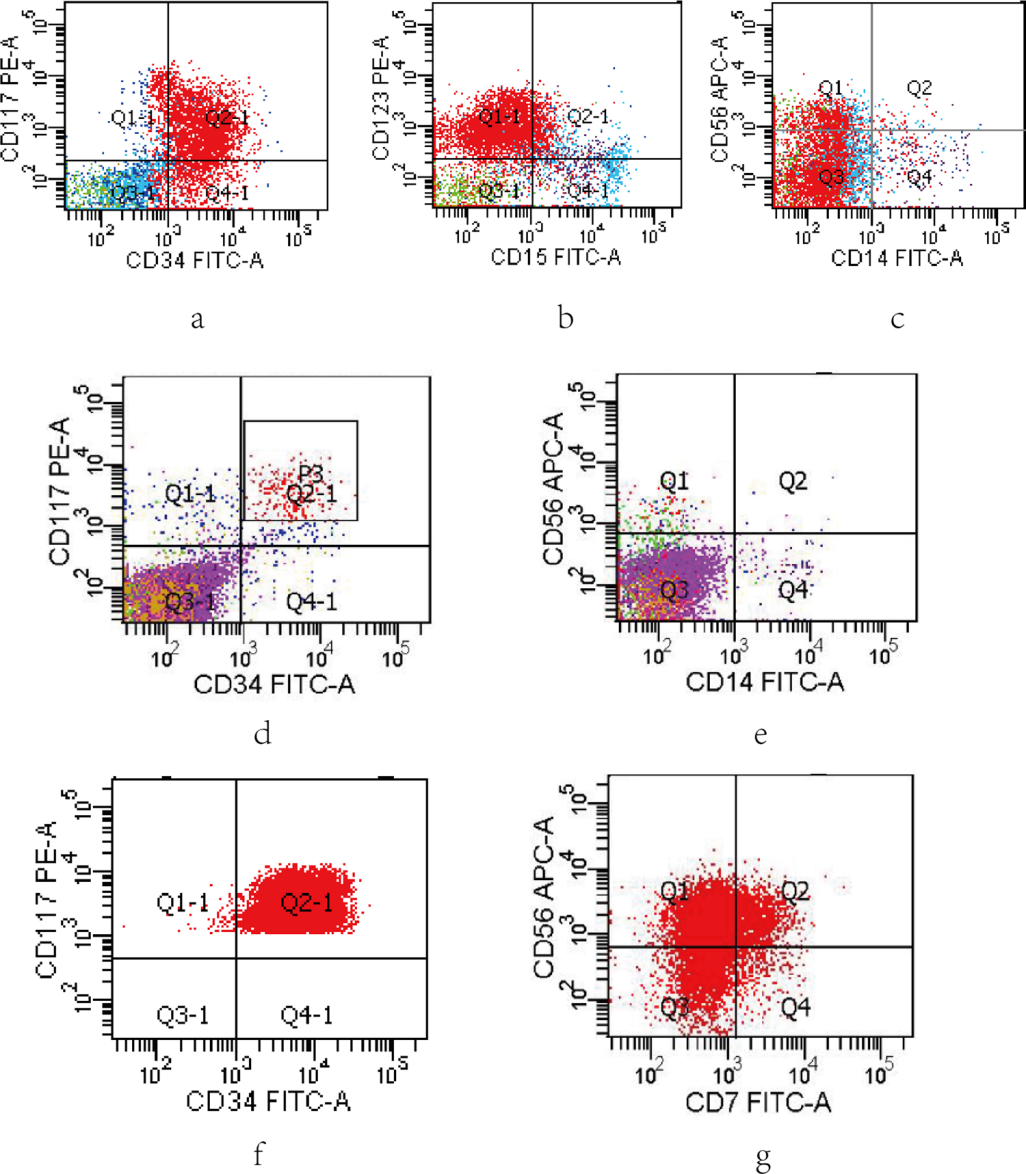
Flow cytometry. **a**, **b**, **c** Flow cytometry result for case 2: mainly expressed CD34, CD117, CD123; partially expressed CD56. **d**, **e** Flow cytometry result for case 3 (the stage of MDS): mainly expressed CD34, CD117; partially expressed CD56. **f**, **g** Flow cytometry result for case 3 (the stage of AML): mainly expressed CD34, CD117, and CD56

**Fig. 4:**
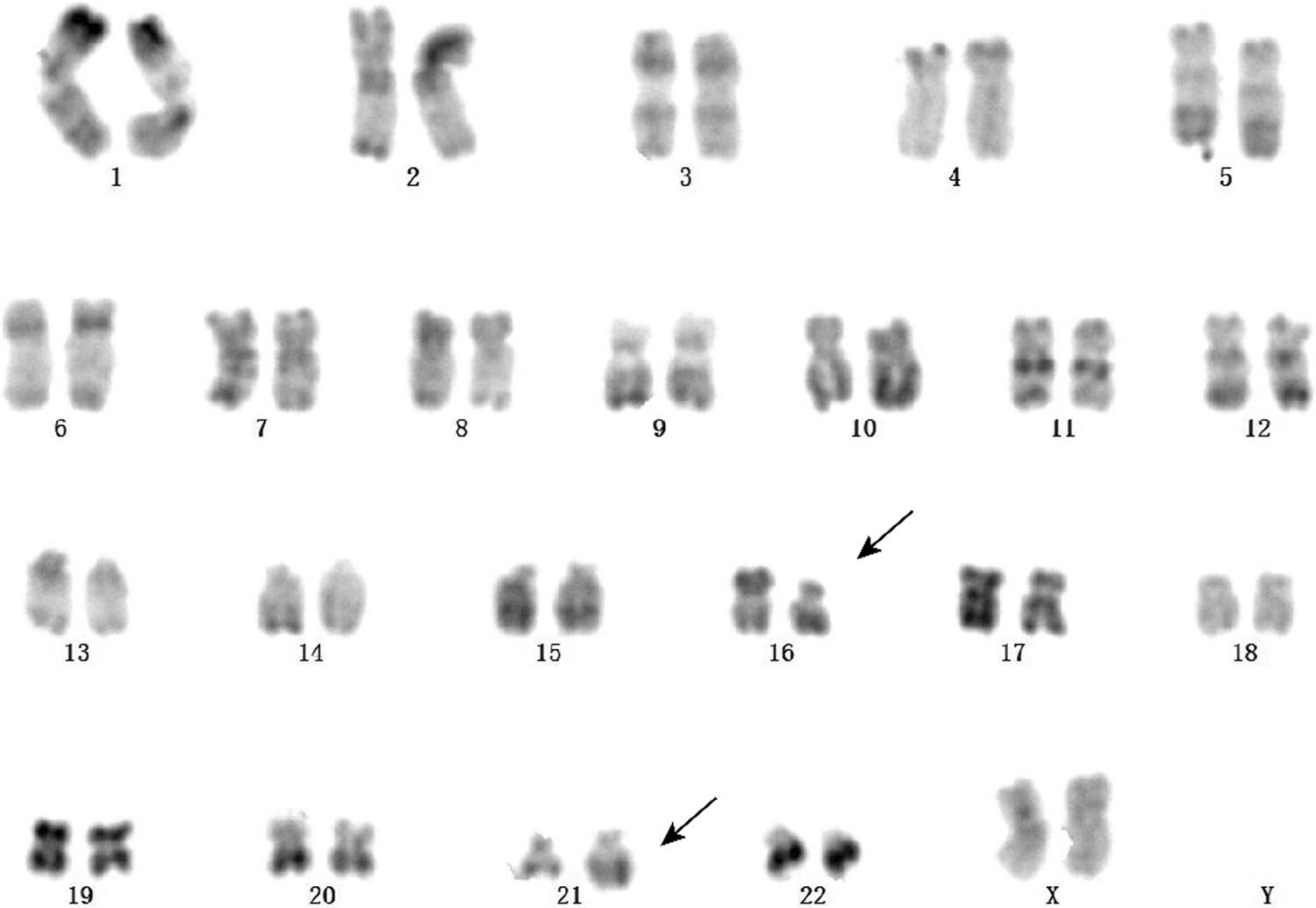
Chromosomal abnormality. The karyotype result was 46XX,t(16;21)(p11;q22) of case 2

A 28-year-old female presented with hand joint pain was admitted to our hospital in July 2020. The blood routine examination showed a WBC count of 12.24 × 10^9^/L with 50% of blasts, a Hb level of 97 g/L, and a platelet level of 192 × 10^9^/L. The BM aspirate revealed 47.2% of the primitive and naïve monocytes. Flow cytometry showed that the blasts accounted for 52.6%. And these cells mainly expressed CD117, CD33, CD34, CD13, and CD123; partially expressed CD56, CD38, CD11b, and CD11c (Fig. 3a, 3b, 3c). The karyotype result was 46XX,t(16;21)(p11;q22) (Fig. 4), and the TLS-ERG fusion gene was detected through RT-PCR. The NGS identified a mutation of BCOR (NM-001123383:exon4:c.1532-1533insCCTGGGTGGT:p.V511fs). This patient was diagnosed with AML-M5b. Then, one course of DA induction chemotherapy was given, and the disease reached morphological CR. After that, she received three courses of medium-dose cytarabine, and the BM aspirate suggested CR at the morphological level while still residual malignant myeloid immature cells exist at the level of immunology. The TLS-ERG fusion genes were 0.92%, 0%, and 36.79%, respectively. Another induction chemotherapy was given with DA combined with homoharringtonine. Regrettably, the disease relapsed. The BM aspirate showed 7.2% of the primitive and naïve monocytes and 7.03% of the residual malignant myeloid immature cells. Besides, the fusion genes arrived at 100%. In the following treatment, after two courses of decitabine and half dose of CAG (cytarabine, aclacinomycin, granulocyte colony-stimulating factor), the disease morphological remission and recurs again. Due to the poor physical condition of the patient, we gave azacitidine and homoharringtonine treatment, respectively. However, the proportion of malignant cells was increasing. In August 2021, the patient died of a pulmonary infection.

### Case 3

**Fig. 5:**
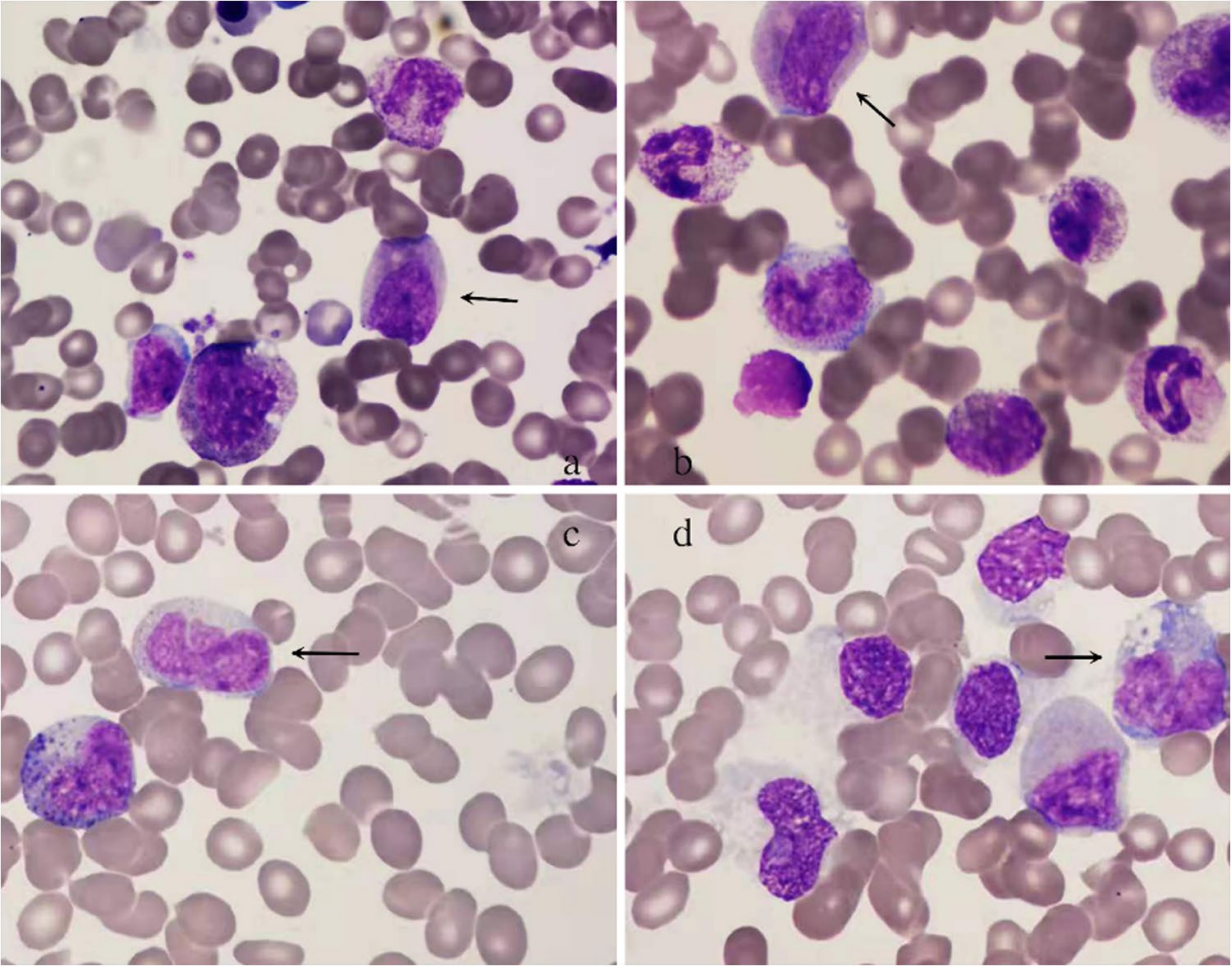
Bone marrow cell morphology of case 3. **a**,** b** MDS RAEB. Both images suggest granulocytic dysplasia. **a** mainly shows the decrease of cytoplasmic particles, and **b** mainly reflects the imbalance of nucleoplasmic development. **c**, **d** MDS progresses to leukemia. Both images suggest an increase in blasts

A 26-year-old male was admitted to our center in May 2019 due to headache and nausea for about 10 days. The initial complete blood cell count revealed a WBC count of 3.87 × 10^9^/L with 3% of blasts, a Hb level of 111 g/L, and a platelet level of 336 × 10^9^/L. The BM aspirate showed 2.8% of the primitive and naïve monocytes (Fig. 5a and 5b), while it was 2% of the primitive and naïve monocytes in the peripheral blood (PB) smear. So, he was diagnosed with MDS-EB-I. Through flow cytometry detection of BM cells, CD33, CD117, and CD34 were mainly expressed; CD123 and CD56 were partially expressed (Fig. 3d and 3e). Conventional cytogenetic analysis and fluorescent in situ hybridization (FISH) were performed, resulting in chromosomal aberration with 47,XY, + 8 t(16;21) (p11;q22) [1]/ 46,XY. Additionally, 27.7% of TLS-ERG fusion genes were confirmed by RT-PCR. And there were no related genetic mutations. He was recommended for allogeneic HSCT (allo-HSCT), but his family member refused. One course of decitabine and half dose of CAG was given then, and the patient developed a scrotal fistula with infection. After that, the patient accepted surgery at the department of proctology without regular chemotherapy. Five months later, the morphology of BM cells showed 44% of the primitive naive monocytes, suggesting the disease had progressed to AML (Fig. 5c and 5d). New gene mutations were detected: nonsense GATA2 (NM_001145661:exon3:c.C71G:p.S24X) and SMAD4 (NM_005359:exon3:c.A262T:p.K88X). Flow cytometry showed 37.66% of the malignant myeloid immature cells. And these cells mainly expressed CD117, CD33, CD34, and CD56 (Fig. 3f and 3g). In addition, the TLS-ERG fusion genes arrived at 110%. Then, we gave this patient decitabine combined with a full dose of CAG, and the patient’s BM morphology achieved CR. Meanwhile, the TLS-ERG sharply decreased to 0.32% without malignant immature myeloid cells can be detected. In March 2020 and May 2020, homoharringtonine was added besides the median dose of cytarabine. Unfortunately, the incidence of relapse was observed in June 2020. The expression of TLS-ERG went up to 82.09%. Then, he received chemotherapy with fludarabine and cytarabine and died in December 2020.

### Case 4

A 27-year-old male was admitted to our hospital in December 2019. The blood routine examination showed a WBC count of 21.44 × 10^9^/L with 85% of blasts and a Hb level count of 82%. Through flow cytometry detection of BM cells, CD117, CD33, CD34, CD13, and CD56 were mainly expressed, while CD11b and CD123 were partially expressed. The karyotype result was 46XY,4q + ,t(16;21)(p11;q22)/45,XY,der(15),t(16;21),-18, and the TLS-ERG fusion gene was detected through RT-PCR. This patient was not sequenced. He was diagnosed with AML-M5, and a standard chemotherapy regimen DA was given. The BM aspirate indicated morphological remission, while still 23.67% of TLS-ERG genes existed. We gave induction chemotherapy DA again, but the fusion genes arrived at 100%. At the third chemotherapy, he received a medium-dose cytarabine regimen. Two months later, the primitive and naïve monocytes count in bone marrow arrived at 74.4%, indicating the incidence of relapse. A HA (homoharringtonine, cytarabine) induction regimen was given again, but the patient eventually died in July 2020.

### Case 5

A 50-year-old male was admitted to our center in March 2018 because of knee pain. The initial complete blood cell count revealed a WBC count of 104.28 × 10^9^/L, a Hb level of 84 g/L, and a platelet level of 31 × 10^9^/L. The BM morphology indicated AML-M5 with 92% of the primitive and naïve monocytes. Through flow cytometry detection of BM cells, CD33, CD117, CD34, CD123, CD56, and CD9 were mainly expressed, while MPO was partially expressed. Karyotype presenting 47,XY, + 8,t(16;21)(p11;q22)/47,XY, + 14,t(16;21)(p11;q22)/46,XY,t(16;21)(p11;q22)del(17). The NGS identified a mutation of KRAS (NM-002524:exon3:c.C181A:p.Q61Krs121913254). After a standard inducing chemotherapy of IA (idarubicin, cytarabine), BM morphology of the patient achieved CR while the fusion gene is still positive. Again, we gave the patient a DA induction regimen, but he died of a pulmonary infection on April 28, 2018.

### Case 6

A 55-year-old male presented with back pain and was admitted to our hospital in December 2017. The blood routine examination showed a WBC count of 83.12 × 10^9^/L, a Hb level of 61 g/L, and a platelet level of 49 × 10^9^/L. The BM aspirate revealed 92% of the primitive and naïve monocytes. Flow cytometry showed the blasts accounted for 88.5%. And these cells mainly expressed CD33, CD117, CD34, CD13, CD123, CD56, and CD4; partially expressed CD38, MPO, and CD11c. The karyotype result was 49,XY, + 8, + 10, + 12, t(16;21) (p11;q22), and the TLS-ERG fusion gene was detected through RT-PCR. In addition, the NGS identified mutations of KRAS (NM-004985:exon2:c.G34T:p.g12c RS 121,913,530) and GATA2 (NM-032638:exon2:c.C106T:p.P36S). He was diagnosed with AML and a chemotherapy regimen HA was given. Regrettably, the patient lost follow-up.

## Results

There are six newly diagnosed patients (five males, one female) with a median age of 39 (26–55) years. The median WBC count is 21.275 (3.87–104.28) × 10^9^/L. Concerning blasts, the median percentage in PB is 67.5 (3–93.9) % and in BM is 70 (2.8–92) %. It is worth noting that three cases (cases 2, 5, 6) show bone and arthrosis pain. Although this kind of pain is one of the clinical manifestations of myeloid leukemia, it is uncommon. For morphology, three cases were diagnosed with AML-M5 (cases 2, 4, and 5), and when the disease progressed to the leukemia stage, case 3 was M5 as well. In addition to myeloid surface antigen CD117, CD34, CD33, and CD13, all six cases showed positive for CD56 and CD123. For case 3, CD56 was mainly expressed at the AML stage compared to partially express at the MDS stage. Besides, chromosome G banding in six cases was t(16;21)(p11;q22), and five cases showed additional chromosomal abnormalities. Case 1 presented a complex three-way translocation with a cryptic t(16;21) in the form of t(11;16;21). Interestingly, this patient shows a more satisfactory treatment effect to the conventional “3 + 7” regimen than the others and can achieve minimal residual disease (MRD) remission. This phenomenon deserves attention because even a small change can sometimes be significant. What’s more, the TLS/FUS-ERG fusion gene was detected in all patients. Given gene mutation, case 2 had BCOR mutation; case 5 and case 6 had KRAS mutation; case 5 had a concurrent GATA2 mutation. Meanwhile, case 3 had no mutation at the stage of MDS, but GATA2 and SMAD4 mutations were identified when the disease progressed to AML. Among the six cases, case 1 is the only patient who remained in sustained remission after induction and consolidation chemotherapy; three cases (cases 2, 4, and 5) received DA or IA induction chemotherapy, achieving morphological CR; case 3 (at AML stage) also achieved morphological CR with the treatment of decitabine combined with a full dose of CAG; and case 6 was lost follow-up. Altogether, the remission effects are not good. Except for case 1 and case 6, the overall survival (OS) of case 2 to case 5 is 13 months, 19 months, 7 months, and 12 months, respectively. It is a pity that none of the patients underwent HSCT.

## Discussion

The incidence of AML with TLS/FUS-ERG fusion gene is approximately 1% [16]. Cases in this review were classified according to French American British (FAB), and all subtypes except M3 were reported. Age at diagnosis ranged from 8 months to 72 years, with a mean age of 27.5, and most were Asian. The morphologically typical characteristics of TLS/FUS-ERG AML are eosinophilia, micromegakaryocytes, hemophagocytosis, and vacuolation of leukemic cells [16, 26]. However, most cases were not presented with eosinophilia, and it was also not observed in the six patients treated at our hospital. Cytogenetically, CD56, which has been suggested to connect with poor prognosis [19], is a characteristic expression in AML with TLS/FUS-ERG. Additionally, it is worth noting that CD56 is related to extramedullary involvement, hemophagocytosis, and vacuolation of leukemic cells [16]. The change of CD56 from partial expression to main expression in case 3 reflects the malignancy to some extent. And the scrotal fistula of this patient was suspected to be caused by leukemic cell infiltration, but no leukemic cells were found on the fistula smear. Other adverse cell surface antigens include CD13, CD33, CD34, and CD25. Kobayashi et al. [14] found specific expression of CD66c in their patients with TLS/FUS-ERG fusion gene, whereas this surficial antigen was not found in other case reports. In recent years, several articles reported that CD123 expressed in TLS/FUS-ERG-positive AML. The antigen expression of CD123, which is mainly expressed in leukemia stem cells, hints the refractory nature of the disease [35, 18

**Fig. 6:**
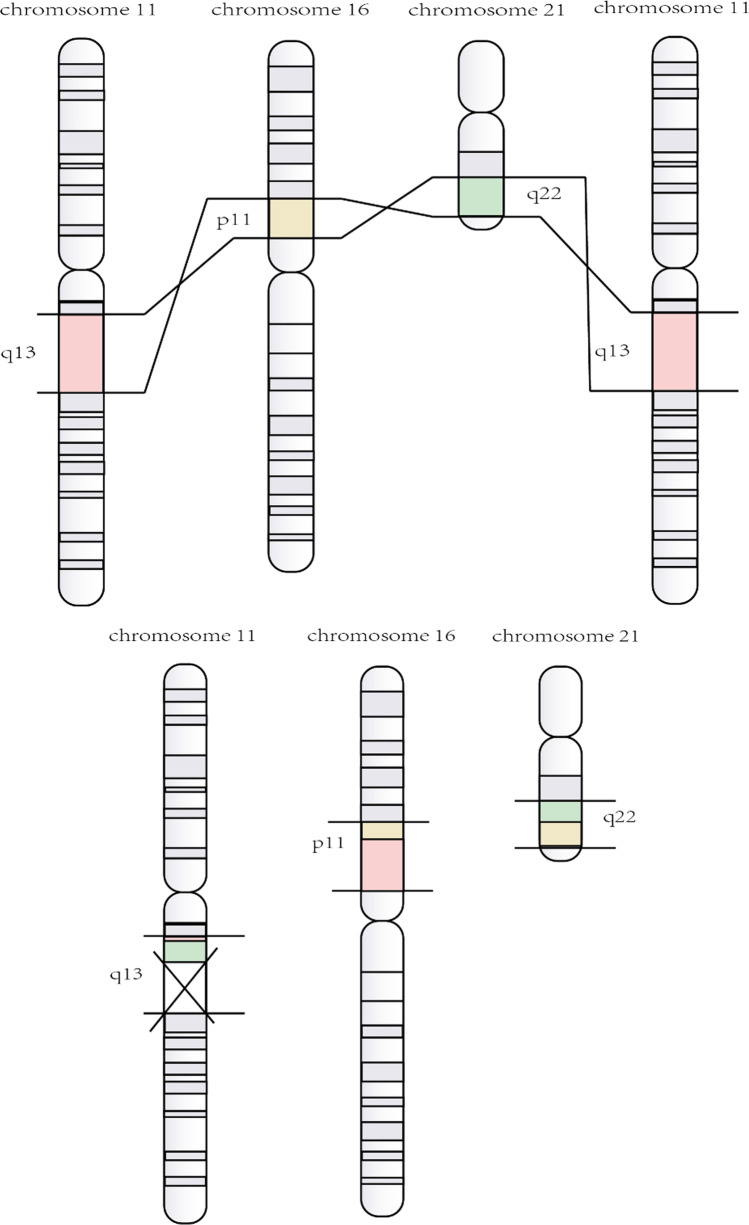
The reciprocal translocation among 11q13, 16p11, and 21q22. The pink part represents q13 on chromosome 11, the yellow part represents p11 on chromosome 16, and the green part represents q22 on chrosomem21. q13 is partially (or wholly) transferred to p11, while p11 is partially (or wholly) transferred to q22. What’s more, q22 is partially (or wholly) transferred to q13

TLS/FUS-ERG fusion gene is produced by t(16;21)(p11;q22) translocation, but some articles discovered unusual jumping translocation in AML with t(16;21) [15, 36]. Jumping translocation is known as a poor prognostic indicator in leukemia and lymphoma [37], involving the nonrandom rearrangements of the chromosome long arm (related to relapse) [38]. However, due to the few reported cases, whether it is involved in the recurrence mechanism remains unclear. In case 1, a novel three-way rearrangement is described whose leukemic cells harbored a variant t(11;16;21). To the best of our knowledge, this is the first case of t(11;16;21)(q13;p11;q22) with a breakpoint at 11q13 (Fig. 6). Three-way translocation is not common. Statistically, variant forms of t(9;22), t(8;21), t(15;17) account for 9.3%, 6.3%, and 2.6%, respectively [39]. At present, the three-way translocation involving 11q23 has been reported more frequently in AML with the MLL fusion gene. Traditionally, chromosomal 11q23 in AML is associated with a poor prognosis. However, per reported cases, patients with MLL variant caused by the three-way translocation, such as t(1;9;11) and t(6;19;11), may have a longer-term remission and a better prognosis than those with the conventional MLL fusion gene [40, 41]. The patient treated at our center remains in complete hematologic remission after induction therapy, which situation is not seen in other patients with t(16;21). And we will continue to clinical follow-up. More broadly, the clinical significance of three-way translocation is still contested because of a small number of cases, and research is also needed.

In another aspect, it is estimated that TLS/FUS-ERG plays a pivotal role in leukemogenesis. Pereira et al. [42] experimented with retroviral transduction of TLS/FUS-ERG in CD34^+^ human hematopoietic cells from cord blood, resulting in a dramatically increased capacity for self-renewal and proliferation of myeloid progenitors. They confirmed that the expression of this fusion gene induced a leukemogenic program. To be more specific, TLS/FUS-ERG affects both the gene transcription and the RNA splicing [21, 43]. Pan et al. [20] demonstrated that compared with progenitor cells harboring empty retroviral vector, terminal differentiation induced by G-CSF was blocked in L-G progenitor cells harboring TLS-ERG. Furthermore, TLS/FUS-ERG-positive L-G cells can proliferate continuously and grow spontaneously without IL-3 in culture. IL-3 is of the essence for mouse L-G myeloid progenitor cells to differentiate into mature neutrophils when treated with G-CSF. In addition, TLS-ERG transformation of L-G myeloid progenitor cells may be related to inhibition of transcription mediated by the fusion protein. As for gene repression by TLS-ERG, the mechanism of repressor complexes involving DNA methylation and histone deacetylation is likely to be critical roles. Interestingly, TLS-ERG transformed L-G cells treated with decitabine or trichostatin A showed retardation of cell growth and recovery of differentiation ability. Besides the uncontrolled cell proliferation and arrest of differentiation, dysregulation of programmed apoptosis is also the reason for tumorigenesis. Ho-keun et al. [25] demonstrated that ERG protein is involved in the inhibition of apoptosis. They treated NIH3T3 cells with calcium ionophore, presenting the obvious phenomenon of apoptosis, while ERG and FUS/TLS-ERG positive cells decreased the level of apoptosis under similar conditions. Meanwhile, ERG and FUS/TLS-ERG inhibit the rate of cell death after the serum withdrawal. TLS/FUS-ERG fusion protein retains the amino-terminal domain of TLS/FUS and the carboxy-terminal domain of ERG. The TLS/FUS fusion domain alters the DNA binding activity and weakens the transcriptional activity of TLS/FUS-ERG chimeric protein [21]. In the above experiments, both TLS/FUS- and ERG-fusion partner domains are indispensable for efficient inhibition of differentiation and apoptosis.

The transcripts have special significance for the type of disease. BCR-ABL is a common fusion gene, and its different transcripts are correlated with disease types. The minor breakpoint cluster region (BCR) rearrangement producing e1a2 fusion transcript is highly associated with Ph^+^ ALL. While e13a2 (b2a2) and/or e14a2 (b3a2) fusion transcript, produced by the major BCR rearrangement, is seen in most cases of CML [44]. For TLS/FUS-ERG, there are four types of chimeric transcripts, with the molecular size of 255, 211, 176, and 349 bp, respectively [35, 45]. Seung [32] has reported an unusual type of transcript (385 bp) in the case of infant ALL, spanning from exon 7 of FUS to exon 6 of ERG. In addition to these, other different types of transcripts have been discovered gradually. In general, it is useful to detect FUS-ERG gene transcripts in monitoring MRD by RT-PCR. According to Noort [11], MRD-positive AML with TLS/FUS‐ERG did not increase the relapse rate. There was no difference in event-free survival (EFS) between MRD-positive and MRD-negative patients, which may be partly due to its an LSC-driven disease. Among the six patients we reported, TLS/FUS-ERG from undetected to reappearing indicates the possibility of relapse, partially explaining the necessity of MRD monitoring. Whether the different transcripts affect disease type and prognosis remains unknown and needs more cases to analyze.

Up to now, seventy-eight mutations have been linked to AML relapse [46]. In TLS/FUS-ERG-positive AML, mutations of several epigenetic regulators can be detected through NGS. It has been reported that RUNX1 mutation can be detected in AML patients with t(16;21)(p11;q22), especially in Southeast Asia [47]. Ismael [22] studied the correlation between RUNX1 mutation and clonal evolution in relapsed AML with t(16;21)(p11;q22). One case of their report presented RUNX1 mutation at the relapse stage, while this mutation was negative at diagnosis, indirectly indicating genetic alterations may play a particular role in relapse pathogenesis. Because of a few cases, it is difficult to draw a firm conclusion about this possible association. In the patients we reviewed, four patients presented RUNX1 mutation; one patient had multiple mutations, including BCOR, PLCG1, DIS3, BRAF, JAK2, and JAK3. In the six cases we reported, one patient had BCOR mutation; two patients had KRAS mutation; one had a concurrent GATA2 mutation; and one occurred with GATA2 and SMAD4 mutations in the stage of disease progression.

HSCT is the preferred alternative for AML patients with high-risk factors. While per Pan et al. [48], allo-HSCT could prolong OS, but may not improve the prognosis of AML harboring t(16;21)(p11;q22). Chimeric antigen receptor (CAR) T cell treatment was performed in a patient expressing CD123, which is one of the significant markers of LSC [23]. The result hints that CAR-T CD123 reduces the chemotherapy-resistant AML blasts. Furthermore, in a multicenter trial, MDS or AML patients at high risk of relapse with MRD-positive can prevent or delay hematological relapse through the regimen of azacytidine [49]. Dai Keino et al. [28] reported a case of pediatric AML with FUS/TLS-ERG, who relapse after allo-HSCT. Then they gave the patient a salvage therapy with azacitidine and achieved the second CR, indicating that it could be a new therapy option. From our therapeutic experience, the traditional “3 + 7” regimen is not enough for patients with t(16;21)(p11;q22) to achieve MRD remission, while “3 + 7 + X” is becoming the consensus for de novo AML treatment. X represents the correspondent drugs to the additional specific changes. For example, midostaurin is added to target FLT3-ITD/TKD mutations; gemtuzumab ozogamicin (GO) is added to target the high expression of CD33; and CPX351 is added to target AML with myelodysplasia-related changes (AML-MRC). If no specific target exists, patients should be treated with a more potent combination chemotherapy regimen.

## Conclusion

In conclusion, TLS/FUS-ERG is not common in AML patients, and most of them are Asian. The characteristics of this disease are a high relapse rate and poor overall prognosis. We found that patients with TLS-ERG are more likely to have bone and arthrosis pain. Expressing CD56 and CD123 and recurring TLS/FUS-ERG may be the signal of refractory and relapse, respectively. Additionally, the existence of jumping translocation was reported in some cases, but the correlation of relapse needs to be confirmed in more cases. We report a novel three-way translocation t(11;16;21)(q13;p11;q22), which may affect the prognosis of patients. AML relapse is also associated with the addition of new mutations and clonal evolution. So, besides monitoring this fusion gene, NGS is also necessary to gain a better understanding of the association between mutations and recurrence. About the pathogenesis of AML with TLS/FUS-ERG, research showed that this fusion gene involves leukemogenesis, differentiation block, and apoptosis resistance. On the molecular level, it affects both gene transcription and RNA splicing. At present, the mechanism still acquaints scarcely, which requires further study.
